# Brief Research Report: The Association Between Educational Experiences and Covid-19 Pandemic-Related Variables, and Mental Health Among Children and Adolescents

**DOI:** 10.3389/fpsyt.2021.647456

**Published:** 2021-04-29

**Authors:** Saray Ramirez, María Paz Aldunate, Carolina Arriagada, Massiel Bueno, Florencia Cuevas, Xaviera González, Ricardo Araya, Jorge Gaete

**Affiliations:** ^1^Departamento de Epidemiología y Estudios en Salud, Facultad de Medicina, Universidad de los Andes, Santiago, Chile; ^2^Office of Children and Adolescents, Municipality of Lo Barnechea, Santiago, Chile; ^3^Health Service and Population Research Department, Institute of Psychiatry, Psychology and Neuroscience, King's College London, London, United Kingdom; ^4^Faculty of Education, Universidad de los Andes, Santiago, Chile; ^5^ANID, Millennium Science Initiative Program, Millennium Nucleus Imhay, Santiago, Chile

**Keywords:** children, adolescents, mental health, wellbeing, pandemic, education

## Abstract

**Introduction:** Mental health problems among children and adolescents are frequent. Today, the world is facing a pandemic with a novel coronavirus, which is related to the higher rates of mental problems reported worldwide. The objective of this study was to determine the impact of the Covid-19 related experiences, educational experiences, and family functioning on mental health and wellbeing among children and adolescents in Chile during the Pandemic and lockdown health measures.

**Methods:** This is a cross-sectional analysis of the first wave of an ongoing longitudinal study among girls and boys of Pre-Kindergarten to 12th grade (4–18 years old) in Santiago, Chile. The sample consisted of 979 students from eight different schools. The method of data collection was online surveys administered to parents and adolescents. The dependent variables were mental health problems and wellbeing. Several independent variables were assessed (sociodemographic variables, Covid-19 related experiences, related educational experiences, and family functioning). A descriptive analysis and univariable and multivariable regression models were performed to study the association between variables.

**Results:** Positive educational experiences, primarily academic self-concept, reduced the probability of mental health problems and increased wellbeing. Among covid-19 related variables, practicing meditation or praying reduced emotional problems, while having family or health problems increased emotional problems among adolescents. No clear association between Covid-19 related experiences variables among children was found.

**Conclusions:** Our findings may help educational and public health authorities to plan future school preventive interventions to improve mental health and wellbeing in this population.

## Introduction

Mental health problems among children and adolescents are frequent and cause important functional deterioration over time (1). Chile is no exception to this burden, especially considering that a third of this population had a diagnosed mental disorder during their childhood and adolescence (2). Furthermore, the world is currently facing a pandemic with a novel coronavirus (SARS-CoV-2), requiring the implementation of prolonged national school closures and remote education in over 107 countries, including Chile (3). Specifically, in Chile, during the whole academic year (March to December 2020), all students had only remote learning experience due to lockdown and school closure policies implemented by the Ministry of Health.

SARS-CoV-2 pandemic has imposed the need to implement several sanitary measures such as lockdowns, school closures, restrictions of outdoor activities, and social distancing, which had dramatically impacted the lives of children and adolescents. For example, they had to rapidly switch from face-to-face learning to remote learning and reduced the interaction with peers. These experiences may have affected their social network, especially in a period of development when social interactions are considered very important. For instance, key aspects of social cognition, including the comprehension of other people's emotions, intentions, and beliefs and the development of new social problem-solving skills, may have been negative impacted due to the sanitary measures during pandemic (4). On the other hand, those students with poor conditions to access remote learning (e.g., lack of computers or smartphones, and restricted internet availability) will probably have a huge negative impact on their academic learning, as several institutions have recently highlighted (5).

Moreover, the SARS-CoV-2 pandemic may have exacerbated already existing high rates of mental health problems among children and adolescents, including emotional and behavioral problems (6–10). Noteworthy, most of the available data on mental health among children and adolescents is coming from developed countries in Europe and North America. It is well-known that lower socioeconomic conditions negatively influence the incidence and prevalence of mental health problems. Therefore, it is expected that the effect of the pandemic on the economy and education will be greater among countries with lower economic development, and especially among low-income families. This is partially explained because disadvantaged families are less likely to have appropriate access to the Internet for remote learning, sufficient living space, and lower opportunities for interaction with peers (11, 12). Additionally, low-income families depend on schools of several supportive measures provided by the government, such as meals, special education, and psychological help for children and adolescents at-risk (8). Having information from developed countries about policies and interventions that may help to improve mental health among children and adolescents may not always be culturally appropriate to be implemented in Low and Middle-Income countries (LMICs), especially among deprived families. This situation creates a gap of knowledge that requires to be fulfilled exploring what is happening in less developed countries (12).

Along with getting information about the prevalence of mental health problems among children and adolescents, it is important to assess the role of related risk and protective factors, which can be found at multiple levels, from individuals to families and the community. Within the community, schools play a key role in providing protective factors on mental health. Studies over the years have found a close relationship between some of these school factors and mental health. For example, a poor self-academic concept has been associated with behavior problems (11), a higher sense of belonging to schools has been related to a reduced risk of mental health problems and have increased prosocial behaviors (12–16). Moreover, higher academic motivation has increased wellbeing and decreased internalizing problems (17, 18). Finally, in the context of the SARS-CoV-2 pandemic, some studies have found that educational factors have been negatively impacted by lockdown measures, especially the schools closing. For example, one study conducted in China reported that a substantial proportion of students were not comfortable or motivated by online education, and consequently, they did not participate effectively during the pandemic (19). Another study conducted in Italy and Portugal also found that online education was associated with lower students' motivation (20). Sadly, none of the mentioned studies during pandemic have explored the relationship between educational variables and mental health. In addition, today, there is only one study exploring anxiety disorders among children during pandemic in Latin American (21), but no information about other mental health problems and among adolescents.

This study intends to contribute to the knowledge gap of the influence of several risk and protective factors on mental health among children and adolescents during pandemic. Specifically, our hypotheses state that some covid-19 related variables such as fear to become infected, family difficulties (e.g., economic, health and functioning) or coping strategies during the pandemic (e.g., meditate, doing physical exercise) and some educational experiences such as having a lower academic motivation or lower school belonging during the lockdowns, will be associated with higher mental health problems and lower life satisfaction.

## Methodology

This is a cross-sectional analysis of the first wave of an ongoing longitudinal study among Pre-Kindergarten to 12th-grade students, during the pandemic with national lockdowns and remote school learning for all students, in Santiago, Chile. A total of 21 schools were invited to participate in August 2020, and eight schools accepted. We approached the students and their main caregivers via e-mail with previous authorization from school authorities, explaining the aim of the study, how to fill out the online questionnaires, and asking for consent. Written and informed consent was signed by caregivers of all students. Main caregivers of children attending Pre-Kindergarten to 4th grade (*4*° *Básico*) responded to an online survey according to the observation of children's behavior. We collected data from adolescents themselves if they were attending 5th grade (*5*° *Básico*) to 12th grade (*IV Medio*), using a similar online survey. Adolescents were also asked to give their assent before answering the survey. After the first invitation, we looked at how many responses we were receiving each day, and we produce a weekly report of this information to be sent to the schools to encourage them to send more invitations and motivate the students and their families to respond to the survey. This procedure was repeated 3 weeks in a row, and we closed the survey after 4 weeks since the initial invitation.

Ethical approval was obtained from the Ethical and Scientific Committee of Universidad de los Andes, Chile (August 20th, 2020; CEC202069).

### Measurements

Dependent variables were mental health problems and general wellbeing. Mental health problems were assessed among children (parent version) and adolescents (students version) using the Strengths and Difficulties Questionnaire (SDQ) (22). This questionnaire is a screening tool for emotional and behavioral problems, which help to detect and assess mental health concerns or potential mental health disorders. It has 25 items, divided into four difficulties subscales (emotional symptoms, conduct problems, hyperactivity-inattention problems, and peer problems) and one strengths subscale (prosocial behavior). Wellbeing was assessed among adolescents with the Student's Life Satisfaction Scale (23). It has seven items, with a Likert scale (1 = *strongly disagree* to 6 = *strongly agree*). Both scales have been validated in Chile (24, 25).

Independent variables explored four domains: sociodemographic features, educational experiences, covid-19 related experiences, and family functioning.

#### Sociodemographic Features

Sex (0 = boys; 1 = girls), Age, Type of school dependency (0 = Public schools; 1 = Subsidized schools; 2 = Private schools).

#### Educational Experiences

Four variables were measured: (1) Last year self-reported Grade Point Average (GPA). The GPA was categorized into three levels: “Poor,” “Regular,” and “Good.” For further details of the categorization, see Appendix Table A.1, in the Supplementary Material. It is worth mentioning that we only included this variable in the survey of adolescents; among children, this grading system was not always applicable, (2) Academic motivation was measured with the Academic Motivation Scale (26) among adolescents. It has 28 items with a Likert scale (1 = *Does not correspond at all* to 7 = *Corresponds exactly*). In the case of children, this variable was measured with a selection of six questions, adapted from the adolescent instrument using a Likert scale (1 = *Not at all Motivated* to 4 = *Highly Motivated*). A high score means high academic motivation, (3) Academic self-concept was measured with the Chilean-validated version of the Academic Self-Concept Scale (27, 28) among adolescents. It has 13 items with a Likert scale (1 = *strongly disagree* to 5 = *strongly agree*). In the case of children, this variable was measured with a selection of six items adapted from the adolescent instrument. Each item was answered with a Likert scale (1 = *Never* to 5 = *Always*). A high score means a high academic self-concept, and (4) Sense of belonging was measured with the abbreviated version of the Psychological Sense of School Membership (29, 30) among adolescents. This scale includes 13 items with a Likert scale (1 = *strongly disagree* to 5 = *strongly agree*). In the case of children, this variable was measured with a selection of five items adapted from the adolescent instrument. It has five items with the same 5-point Likert scale.

#### Covid-19 Related Experiences

We measured the same variables among children and adolescents. We explored having “Fear to contracting Covid-19” and “Fear that a family member or friend contracts Covid-19,” answering on a 5-point scale (1 = *Not at all* to 5 = *Extremely*). We also measured the frequency, on a 6-point scale (from 1 = “*0 days*” to 6 = “*Everyday*”), of doing the following activities: Socializing online, Doing exercise, Involved in leisure activities, and Meditated and prays. Finally, we asked for the frequency, on a 5-point scale (1 = *None* to 5 = *A lot*), of having the following problems during Pandemic: Financial problems, Family problems, Health problems, and Teaching accessibility problems. In order to simplify the analysis, all these variables were grouped into two categories reflecting low vs. high fear or frequency, accordingly. For more details, see Appendix Table A.1, in the Supplementary Material.

#### Family Functioning

We used the short version of the Family Adaptability and Cohesion Evaluation Scale (FACES-20) (31, 32), which has 20 items with a Likert scale (0 = *Never* to 4 = *Almost always*). A high score means high family adaptability and cohesion.

### Data Analysis

A descriptive analysis was performed with measures of variance by calculating 95% confidence intervals and standard deviation accordantly. Measures of central tendency were calculated with the mean, and finally, to represent relative frequencies, percentages were presented.

Univariable and multivariable regression models were performed (mixed models) in two sequential steps: (1) Unadjusted models: all variables were assessed to determine if they were associated with each of the six outcomes (See all unadjusted results in the Appendix Table A.2, in the Supplementary Material); (2) Adjusted models: for each outcome variable, those factors that had a univariable association (*p* ≤ 0.05), were selected to be included in the final multivariable model. All final models included sex and age as covariates (See Table 2). For interpretation analyses, *p* ≤ 0.05 were considered statically significant, and all the independent variables assessed by scales (e.g., academic motivation) considered the following interpretation: increasing in 1 point of the total scale score would increase or decrease the Beta coefficient (β) of the outcome. All statistical analyses were performed using Stata 15.

## Results

The total number of students eligible for this study was 7,968, but we received information from 979 students (12.3%). All data were collected during SARS-CoV-2 lockdown, with school closures, and all students had remote learning. It is important to highlight that most of the main caregivers who answered the questionnaires (for children from Kindergarten and 4th grade) corresponded to the children's parents (86.6% were mothers, 9.2% were fathers, 2% were grandmothers, 1.7% were other relatives, and 0.5% were aunts). Most participants were girls. Physical exercise, get involved in leisure activities, and meditate or pray were rarely practiced. On the other hand, between 17.5% (Health problems among children) and 52% (Financial problems among children) have had experienced different problems during the pandemic. For further information on descriptive variables, see Table 1.

**Table 1:** Descriptive variables.

**Variables**	**Children**			**Adolescents**		
**Sociodemographic**	***n***	**% or mean**	**[95% CI] or (SD)**	***n***	**% or mean**	**[95% CI] or (SD)**
Sex						
Girls	339	56.3	[52.3–60.2]	224	59.4	[54.4–64.3]
Boys	263	43.7	[39.8–47.7]	153	40.6	[35.7–45.6]
Age	602	8.3	(1.8)	377	15.3	(2.4)
Type of school dependency						
Private	226	37.5	[33.7–41.5]	182	48.3	[43.2–53.3]
Subsidized	320	53.2	[49.1–57.1]	116	30.7	[26.3–35.6]
Public	56	9.3	[7.2–11.9]	79	21.0	[17.1–25.4]
Educational experiences						
Last year self-reported Grade Point Average (GPA)						
Poor	N/A	N/A	N/A	15	4.0	[2.4–6.5]
Regular	N/A	N/A	N/A	131	34.7	[30.1–39.7]
Good	N/A	N/A	N/A	231	61.3	[56.2–66.1]
Academic motivation	560	37.5	(3.9)	377	67.5	(12.5)
Academic self-concept	518	19.5	(4.5)	377	46.2	(8.1)
Sense of belonging	554	22.6	(3.3)	377	53.1	(8.4)
Covid-19 related experiences						
Fear to contracting Covid-19						
No fear	218	36.2	[35.5–40.1]	127	33.7	[29.1–38.6]
Fear	384	63.8	[59.9–67.5]	250	66.3	[61.4–70.9]
Fear that a family member or friend contracts Covid-19						
No fear	176	29.2	[25.7–33.0]	28	7.4	[5.2–10.6]
Fear	426	70.8	[67.0–74.3]	349	92.6	[89.4–94.8]
Socializing online						
Yes	157	26.1	[22.7–29.7]	229	60.7	[55.7–65.6]
No	445	73.9	[70.3–77.3]	148	39.3	[34.4–44.3]
Doing exercise						
Yes	209	34.7	[22.7–29.7]	142	37.7	[32.9–42.7]
No	393	65.3	[70.3–77.3]	235	62.3	[57.3–67.1]
Involved in leisure activities						
Yes	263	43.7	[39.8–47.7]	138	36.6	[31.9–41.6]
No	339	56.3	[52.3–60.2]	239	63.4	[58.4–68.1]
Meditates and prays						
Yes	204	33.9	[30.2–37.8]	71	18.8	[15.2–23.1]
No	398	66.1	[62.2–69.8]	306	81.2	[76.9–84.8]
Financial problems						
Yes	313	52.0	[48.0–56.0]	133	35.3	[30.6–40.3]
No	289	48.0	[44.0–52.0]	244	64.7	[59.7–69.4]
Family problems						
Yes	162	26.9	[23.5–30.6]	119	31.6	[27.1–36.5]
No	440	73.1	[69.4–76.5]	258	68.4	[63.5–72.9]
Health problems						
Yes	105	17.5	[14.6–20.7]	74	19.6	[15.9–24.0]
No	496	82.5	[79.3–85.4]	303	80.4	[76.0–84.1]
Teaching accessibility problems						
Yes	188	31.2	[27.6–35.1]	119	31.6	[27.1–36.5]
No	414	68.8	[65.0–72.4]	258	68.4	[63.5–72.9]
Family functioning						
FACES-20 scale	596	63.2	(10.8)	377	58.4	(14.7)
Mental Health and Wellbeing						
SDQ subscales						
Emotional symptoms	602	0.5	(0.9)	377	1.0	(1.3)
Conduct problems	602	1.8	(0.8)	377	1.7	(0.8)
Hyperactivity problems	602	4.2	(1.4)	377	4.1	(1.4)
Peer problems	602	2.8	(0.9)	377	2.9	(1.0)
Prosocial behavior	602	3.4	(1.4)	377	3.2	(1.5)
Life satisfaction	N/A	N/A	N/A	377	32.4	(7.1)

*N/A, not applicable because the variable was not assessed among Children. CI, confidence interval; SD, standard deviation*.

In the adjusted results, we found that when children get older, they reduced conduct, hyperactivity, and peer problems, see Table 2. And among adolescents happened the same phenomenon in conduct and hyperactivity problems, see Table 3. Attending a private school reduced the probability of having hyperactivity problems among children, and peer problems among adolescents. Girls from the children group had a reduced probability of having conduct, hyperactivity and peer problems, and on the other hand, adolescent girls had an increased probability of having emotional symptoms, but at the same time, they had more prosocial skills when compared to boys.

**Table 2:** Adjusted regression models exploring the association between risk and protective factors and mental health problems among children.

**Variables**	**Emotional symptoms**		**Conduct problems**		**Hyperactivity problems**		**Peer problems**		**Prosocial behavior**	
	**β**	***P*-value**	**β**	***P*-value**	**β**	***P*-value**	**β**	***P*-value**	**β**	***P*-value**
**Sociodemographic**										
Sex^a^										
Girls	0.01	0.921	0.07	0.357	−0.08	0.499	−0.1	0.264	0.10	0.420
Age	−0.00	0.965	−0.05	0.022^*^	−0.1	0.002^*^	−0.07	0.010^*^	0.01	0.781
**Type of school dependency**^**b**^										
Subsidized	N/A	N/A	N/A	N/A	−0.25	0.219	0.18	0.220	N/A	N/A
Private	N/A	N/A	N/A	N/A	−0.69	0.002^*^	−0.2	0.212	N/A	N/A
**Educational experiences**										
Academic motivation	−0.02	0.057	−0.06	0.000^**^	−0.09	0.000^**^	−0.01	0.539	0.09	0.000^**^
Academic self-concept	−0.07	0.000^**^	−0.03	0.012^*^	−0.09	0.000^**^	−0.04	0.003^*^	0.00	0.993
Sense of belonging	−0.01	0.424	−0.01	0.622	−0.02	0.265	−0.02	0.105	0.03	0.170
**Covid-19 related experiences**										
Fear to contracting Covid-19	0.15	0.154	N/A	N/A	N/A	N/A	N/A	N/A	N/A	N/A
Fear that a family member or friend contracts Covid-19	−0.01	0.930	N/A	N/A	N/A	N/A	N/A	N/A	N/A	N/A
Socializing online	N/A	N/A	N/A	N/A	N/A	N/A	−0.07	0.451	N/A	N/A
Doing exercise	N/A	N/A	0.01	0.911	N/A	N/A	0.03	0.728	0.01	0.969
Involved in leisure activities	N/A	N/A	0.01	0.903	0.08	0.502	0.00	0.984	0.2	0.132
Meditates and prays	N/A	N/A	−0.1	0.172	N/A	N/A	N/A	N/A	0.25	0.055
Financial problems	−0.01	0.888	0.11	0.155	0.00	0.999	−0.06	0.571	N/A	N/A
Family problems	0.15	0.104	−0.06	0.515	N/A	N/A	0.19	0.077	−0.01	0.919
Health problems	0.01	0.884	N/A	N/A	0.00	0.999	0.12	0.282	N/A	N/A
Teaching accessibility problems	−0.07	0.400	0.02	0.788	−0.1	0.495	0.04	0.723	N/A	N/A
**Family functioning**										
FACES-20 scale	−0.01	0.055	−0.01	0.005^*^	−0.00	0.404	−0.01	0.011^*^	0.03	0.000^**^

*All models were adjusted by sex, age, and variables associated (p < 0.05) with mental health and wellbeing outcomes in the univariable regression models (See Appendix Table A.2, in the Supplementary Material). N/A, not applicable because the variable was not associated in the unadjusted models*.

a *Sex [0 = Boys (Ref); 1 = Girls]*;

b *Type of school dependency [0 = Public (Ref); 1 = Subsidized; 2 = Private]*.

* *p ≤ 0.05*

** *p ≤ 0.001*.

**Table 3:** Adjusted regression models exploring the association between risk and protective factors and mental health problems and well-being among adolescents.

**Variables**	**Emotional symptoms**		**Conduct problems**		**Hyperactivity problems**		**Peer problems**		**Prosocial behavior**		**Life satisfaction**	
	**β**	***P*-value**	**β**	***P*-value**	**β**	***P*-value**	**β**	***P*-value**	**β**	***P*-value**	**β**	***P*-value**
**Sociodemographic**												
**Sex**^**a**^												
Girls	0.49	0.000^**^	−0.26	0.000^**^	−0.09	0.493	0.1	0.263	0.41	0.004^*^	−0.95	0.107
Age	0.01	0.686	−0.05	0.002^*^	−0.08	0.006^*^	−0.00	0.889	0.06	0.066	0.01	0.951
**Type of school dependency**^**b**^												
Subsidized	N/A	N/A	0.16	0.134	N/A	N/A	−0.01	0.968	−0.47	0.016^*^	0.33	0.688
Private	N/A	N/A	−0.21	0.062	N/A	N/A	−0.36	0.010^*^	0.33	0.120	1.28	0.151
**Educational experiences**												
Last year self-reported Grade Point Average (GPA)^c^												
Regular	N/A	N/A	−0.3	0.116	−0.51	0.141	0.05	0.820	0.12	0.730	3.33	0.027^*^
Good	N/A	N/A	−0.37	0.063	−0.47	0.170	−0.06	0.807	0.19	0.609	3.65	0.022^*^
Academic motivation	−0.00	0.993	−0.01	0.005^*^	−0.01	0.207	−0.01	0.315	0.02	0.000^**^	0.09	0.001^**^
Academic self-concept	−0.04	0.000^**^	−0.02	0.001^*^	−0.07	0.000^**^	−0.02	0.001^**^	0.00	0.651	0.15	0.000^**^
Sense of belonging	−0.03	0.005^*^	−0.00	0.413	0.01	0.556	−0.02	0.000^**^	0.03	0.008^*^	0.12	0.007^*^
Covid-19 related experiences												
Fear to contracting Covid-19	0.26	0.058	N/A	N/A	N/A	N/A	N/A	N/A	0.01	0.964	N/A	N/A
Fear that a family member or friend contracts Covid-19	0.57	0.017^*^	N/A	N/A	N/A	N/A	N/A	N/A	N/A	N/A	N/A	N/A
Socializing online	N/A	N/A	N/A	N/A	N/A	N/A	−0.29	0.002^*^	0.28	0.044^*^	N/A	N/A
Doing exercise	N/A	N/A	N/A	N/A	N/A	N/A	−0.1	0.275	0.09	0.523	0.12	0.837
Involved in leisure activities	N/A	N/A	N/A	N/A	N/A	N/A	N/A	N/A	0.04	0.761	−0.01	0.985
Meditates and prays	−0.35	0.027^*^	N/A	N/A	−0.22	0.210	N/A	N/A	0.57	0.002^*^	−0.5	0.471
Financial problems	N/A	N/A	N/A	N/A	N/A	N/A	0.07	0.472	N/A	N/A	−0.72	0.251
Family problems	0.29	0.032^*^	N/A	N/A	0.31	0.043^*^	N/A	N/A	N/A	N/A	−0.89	0.108
Health problems	0.44	0.003^*^	N/A	N/A	N/A	N/A	0.13	0.261	N/A	N/A	−2.65	0.000^**^
Teaching accessibility problems	N/A	N/A	N/A	N/A	0.11	0.463	−0.01	0.896	N/A	N/A	−0.91	0.153
**Family functioning**												
FACES-20 scale	−0.00	0.509	−0.00	0.143	−0.00	0.350	0.00	0.790	0.24	0.125	0.14	0.000^**^

*All models were adjusted by sex, age, and variables associated (p < 0.05) with mental health and wellbeing outcomes in the univariable regression models (See Appendix Table A.2, in the Supplementary Material). N/A, not applicable because the variable was not associated in the unadjusted models*.

a *Sex [0 = Boys (Ref); 1 = Girls]*;

b *Type of school dependency [0 = Public (Ref); 1 = Subsidized; 2 = Private]*;

c *Last year self-reported Grade Point Average [0 = Poor (Ref); 1 = Regular; 2 = Good]*.

* *p ≤ 0.05*

** *p ≤ 0.001*.

Among children, higher academic motivation was associated with a lower probability of experiencing conduct and hyperactivity problems and an increased probability of reporting prosocial behavior. In adolescents, higher motivation reduced the probability of having conduct problems and an increased probability of having prosocial behaviors and higher life satisfaction. Academic self-concept reduced the probability of all four mental health problems in both children and adolescents and increased the probability of higher life satisfaction among adolescents. Sense of belonging reduced the probability of having emotional and peer problems and an increased probability of having prosocial skills and higher life satisfaction among adolescents.

No clear association between Covid-19 related experiences variables among children was found. However, among adolescents who reported having the fear that a family member or friend could contracts Covid-19, having family and health problems during the pandemic increased the probability of having emotional symptoms. On the contrary, adolescents who reported being involved frequently in meditation and prayer reduced the probability of having emotional symptoms. Moreover, those adolescents who reported having more activities of socializing online and meditation and prayer increased the probability of prosocial behavior.

Finally, among children, a higher family functioning reduced the probability of conduct problems and peer problems and increased the probability of prosocial behavior; and among adolescents, it increased the probability of higher life satisfaction. See Tables 1, 2.

## Discussion

To our knowledge, this is the first study in Latin America that assessed the impact of different risk and protective factors, such as, Covid-19 related experiences, educational variables, and family functioning, under the context of school closures, remote learning, and lockdowns throughout the whole academic year in Chile, on mental health problems and wellbeing among children and adolescents.

Our findings highlight that potential modifiable educational variables, such as academic motivation, academic self-concept, and sense of belonging to the school, may be used in preventive interventions not only to increase academic performance but to improve mental health and wellbeing among children and adolescents. Similar contexts to Chile may see these results as informative and useful to plan their own interventions. It is clear the necessity of preventive interventions worldwide (33) to reduce the impact of the SARS-CoV-2 pandemic in the life of children, adolescents, and their families.

Under the pandemic context, there is little previous research about these educational experiences. These studies did not associate their findings with mental health, but we think it is important to highlight that they found a substantial proportion of students who are not comfortable or motivated by online education (19, 20) and consequently, we can conclude that they may develop or increase mental health problems if we contrast this with our results, which is worrying.

Other previous studies have assessed mental health problems among children and adolescents under the SARS-CoV-2 pandemic context, also using the SDQ questionnaire as a screening tool. For example, one Indonesian report (34) found that among adolescents, poor parental support increased the total difficulties score of the SDQ and decreased prosocial behavior. In our research, using a positive dimension of family functioning, we found that better functioning was associated with higher scores on the prosocial behavior subscale among children, and higher wellbeing among adolescents. In a similar way, another study in Italy found that having difficulties in the management of the parent–child relationship during covid-19 quarantine increased the presence of emotional symptoms, hyperactivity, and conduct problems among children and adolescents (35).

Regarding some demographic variables, we found higher prosocial behavior among adolescent girls, similar to a study conducted in Italy (7), where they also found these results among children. Finally, a study in Germany found higher rates of all problems and symptoms assessed by SDQ among children and adolescents coming from low socioeconomic status and of parents with lower education (36). We also found that children of low-income families (attending public schools) had a higher risk of hyperactive problems, and adolescents coming from low-income families (attending public schools) had a higher risk of peer problems when compared with students of private schools, usually of affluent families.

Regarding Covid-19-related experiences, we found that they seem to have a significant impact on adolescents, partially similar to other studies where adolescents and children have had a negative impact (9, 10). Furthermore, in contrast to our findings, a recent review (8) highlights a higher impact on children, specifically in the topic of having the fear that a family member could contract Covid-19.

Among the strengths of this study, we can mention that participating schools were representative of three types of school dependencies with different socioeconomic backgrounds. We also used valid instruments to measure educational and mental health variables. Additionally, we included several educational indicators in the analyses as independent variables, which are not always included in studies of mental health. Finally, our results may contribute to providing information on risk and protective factors for mental health, especially for countries with similar characteristics like Chile, which could help to implement preventive interventions in schools.

Some potential limitations are related to the fact that this was a cross-sectional analysis where no causality conclusion can be made. Due to the fact that we had a higher proportion of girls participating in the study, a potential gender bias may have been introduced in the results. In future data collection, this issue should be considered carefully, and measures to assure equal participation should be implemented. Additionally, all questionnaires were self-reported; consequently, participants may have introduced desirability bias. In addition, the impossibility of applying the same instruments to measure some of the variables among children and adolescents, and the fact that we have different informants between groups (parents or caregivers for children and adolescents by themselves) may reduce the possibility of comparing the results of children and adolescents. Low participation may reduce the generalization of the results. It is worth mention that this issue may have happened because the survey was conducted in the last trimester of the 2020 academic year in Chile. Due to all the changes and adaptations that schools implemented during the whole year, especially moving the education from face to face to remote learning experience, school staff, parents, and students may have been exhausted and less motivated to participate in our study. Additionally, we were not able to compare these results with students who were not experiencing the lockdown measures or the closing of the schools, because all primary and secondary students had the same experience during the whole academic year in Chile. And finally, the mental health problems outcomes measured in this study should be interpreted carefully, because the instrument used here (SDQ) is not a diagnostic tool but a screening tool, and therefore, no mental disorders could be clearly detected.

Our research provides useful information about risk and protective factors that may be modifiable such as academic motivation, academic self-concept, and school belonging. This information may help educational and public health authorities to plan future school preventive interventions to improve mental health and wellbeing in this population.

## Data Availability Statement

The raw data supporting the conclusions of this article will be made available by the authors, without undue reservation.

## Ethics Statement

Ethical approval was obtained from the Ethical and Scientific Committee of Universidad de los Andes, Chile (August 20th, 2020; CEC202069). Written informed consent to participate in this study was provided by the participants' legal guardian/next of kin.

## Author Contributions

JG: conceptualization, methodology, validation, formal analysis, investigation, data curation, writing—review and editing, visualization, supervision, project administration, and funding acquisition. SR: software, formal analysis, writing—original draft. MA, CA, MB, FC, and XG: resources. RA: writing—review and editing. All authors contributed to the article and approved the submitted version.

## Conflict of Interest

The authors declare that the research was conducted in the absence of any commercial or financial relationships that could be construed as a potential conflict of interest.
