# Supplementary material for: Brief Research Report: The Association Between Educational Experiences and Covid-19 Pandemic-Related Variables, and Mental Health Among Children and Adolescents
Source: Front Psychiatry. 2021 Apr 29;12:647456. doi: 10.3389/fpsyt.2021.647456 (PMC8116561; doi:10.3389/fpsyt.2021.647456)
Supplement: Supplementary file 1 [file Data_Sheet_1.docx]

Appendix Table A.1: Variables categorization

| **Variable** | **Item for Children** | **Item for Adolescents** | **Original categorization** | **Distribution for simplified categorization** | **Final categorization** |
| --- | --- | --- | --- | --- | --- |
| Last year self-reported Grade Point Average (GPA). The grading Chilean system goes from 1 to 7. | Not applicable | Your Last year self-reported Grade Point Average (GPA) was.. | Less than 4.0 = 1  Between 4.0-4.4 = 2  Between 4.5-4.9 = 3  Between 5.0-5.4 = 4  Between 5.5-5.9 = 5  Between 6.0-6.4 = 6  Between 6.5-7.0 = 7 | Poor = “Less than 4.0”, “Between 4.0-4.4 and “Between 4.5-4.9”  Regular = “Between 5.0-5.4” and “Between 5.5-5.9”  Good = “Between 6.0-6.4” and “Between 6.5-7.0” | Poor = 0  Regular = 1  Good = 2 |
| Fear to contracting Covid-19 | Does your child or child in your care have a fear or concern of contracting coronavirus (Covid-19)? | Are you afraid or worried about contracting coronavirus (Covid-19)? | Not at all = 1  A little = 2  Somewhat = 3  Much = 4  Extremely = 5 | No, he/she is not afraid = “Nothing” and “A little”  Yes, he/she is afraid = “Somewhat”, “Much” and “Too much” | No, he/she is not afraid = 0  Yes, he/she is afraid = 1 |
| Fear that a family member or friend contracts Covid-19 | Does your child or child in your care have a fear or concern that a family member or friend contracts coronavirus (Covid-19)? | Are you afraid or worried that a family member or friend will contract  coronavirus (covid-19)? | Not at all = 1  A little = 2  Somewhat = 3  Much = 4  Extremely = 5 | No, he/she is not afraid = “Nothing” and “A little”  Yes, he/she is afraid = “Somewhat”, “Much” and “Too much” | No, he/she is not afraid = 0  Yes, he/she is afraid = 1 |
| Socializing online | During this period of sanitary measures. How often does your child or child in your care socialized online (without  school purposes)? | During this period of sanitary measures. How often do you socialize online (without  school purposes)? | 0 days = 1  One time at a month or less = 2  1-2 days a week = 3  3-4 days a week = 4  5-6 days a week = 5  Everyday = 6 | No, he/she has not socialized online = “0 days”, “One time at a month or less” and “1-2 days a week”  Yes, he/she has socialized online = “3-4 days a week”,  “5-6 days a week” and “Everyday” | No, he/she has not socialized online = 0  Yes, he/she has socialized online = 1 |
| Doing exercise | During this period of sanitary measures. How often does your child or child in your care exercise? | During this period of sanitary measures. How often do you exercise? | 0 days = 1  One time at a month or less = 2  1-2 days a week = 3  3-4 days a week = 4  5-6 days a week = 5  Everyday = 6 | No, he/she has not done exercise = “0 days”, “One time at a month or less” and “1-2 days a week”  Yes, he/she has done exercise = “3-4 days a week”,  “5-6 days a week” and “Everyday” | No, he/she has not done exercise = 0  Yes, he/she has done exercise = 1 |
| Involved in leisure activities | During this period of sanitary measures. How often does your child or child in your care get involved in leisure activities? | During this period of sanitary measures. How often are you involved in leisure activities? | 0 days = 1  One time at a month or less = 2  1-2 days a week = 3  3-4 days a week = 4  5-6 days a week = 5  Everyday = 6 | No, he/she is not involved in leisure activities = “0 days”, “One time at a month or less” and “1-2 days a week”  Yes, he/she has is involved in leisure activities = “3-4 days a week”,  “5-6 days a week” and “Everyday” | No, he/she is not involved in leisure activities = 0  Yes, he/she is involved in leisure activities = 1 |
| Meditated and prays | During this period of sanitary measures. How often does your child or child in your care meditated or prays? | During this period of sanitary measures. How often do you meditate or pray? | 0 days = 1  One time at a month or less = 2  1-2 days a week = 3  3-4 days a week = 4  5-6 days a week = 5  Everyday = 6 | No, he/she has not done meditation or prayed = “0 days”, “One time at a month or less” and “1-2 days a week”  Yes, he/she has done meditation or prayed = “3-4 days a week”,  “5-6 days a w eek” and “Everyday” | No, he/she has not done meditation or prayed = 0  Yes, he/she has done meditation or prayed = 1 |
| Financial problems | During the quarantine period, did your family have financial problems due to the  coronavirus situation  (Covid-19)? | During the quarantine period, did your family have financial problems due to the  coronavirus situation  (Covid-19)? | None = 1  A few = 2  Some = 3  Many = 4  A lot = 5 | No, he/she has not financial problems = “Nothing” and “A few”  Yes, he/she has financial problems = “Some”, “Many” and “Too Many” | No, he/she has not financial problems = 0  Yes, he/she has financial problems = 1 |
| Family problems | During the quarantine period, did your family have family problems (  arguments, fights, conflicts) due to the  coronavirus situation  (Covid-19)? | During the quarantine period, did your family have family problems (  arguments, fights, conflicts) due to the  coronavirus situation  (Covid-19)? | None = 1  A few = 2  Some = 3  Many = 4  A lot = 5 | No, he/she has not family problems = “Nothing” and “A few”  Yes, he/she has family problems = “Some”, “Many” and “Too Many” | No, he/she has not family problems = 0  Yes, he/she has family problems = 1 |
| Health problems | During the quarantine period, did your family have health problems (Covid-19 or any other disease) due to the  coronavirus situation  (Covid-19)? | During the quarantine period, did your family have health problems (Covid-19 or any other disease) due to the  coronavirus situation  (Covid-19)? | None = 1  A few = 2  Some = 3  Many = 4  A lot = 5 | No, he/she has not health problems = “Nothing” and “A few”  Yes, he/she has health problems = “Some”, “Many” and “Too Many” | No, he/she has not health problems = 0  Yes, he/she has health problems = 1 |
| Teaching accessibility problems | During the quarantine period, did your family have teaching accessibility problems due to the  coronavirus situation  (Covid-19)? | During the quarantine period, did your family have teaching accessibility problems due to the  coronavirus situation  (Covid-19)? | None = 1  A few = 2  Some = 3  Many = 4  A lot = 5 | No, he/she has not teaching accessibility problems = “Nothing” and “A few”  Yes, he/she has teaching accessibility problems = “Some”, “Many” and “Too Many” | No, he/she has not teaching accessibility problems = 0  Yes, he/she has teaching accessibility problems = 1 |
