# Supplementary material for: Brief Research Report: The Association Between Educational Experiences and Covid-19 Pandemic-Related Variables, and Mental Health Among Children and Adolescents
Source: Front Psychiatry. 2021 Apr 29;12:647456. doi: 10.3389/fpsyt.2021.647456 (PMC8116561; doi:10.3389/fpsyt.2021.647456)
Supplement: Supplementary file 2 [file Data_Sheet_2.docx]

Table A.2: Univariable associations

| Variables | Emotional symptoms | | Conduct problems | | Hyperactivity problems | | Peer problems | | Prosocial behavior | | Life Satisfaction |
| --- | --- | --- | --- | --- | --- | --- | --- | --- | --- | --- | --- |
|  | Children | Adolescents | Children | Adolescents | Children | Adolescents | Children | Adolescents | Children | Adolescents | Adolescents |
| Sociodemographic |  |  |  |  |  |  |  |  |  |  |  |
| Sex |  |  |  |  |  |  |  |  |  |  |  |
| *Boys* | 1 | 1 | 1 | 1 | 1 | 1 | 1 | 1 | 1 | 1 | 1 |
| *Girls* | -0.07  0.365 | 0.53  **0.000 | -0.02  0.767 | -0.32  **0.000 | -0.21  0.079 | -0.15  0.297 | -0.15  0.057 | 0.07  0.489 | 0.20  0.084 | 0.55  **0.001 | -0.65  0.379 |
| Age | 0.02  0.273 | 0.13  *0.000 | -0.03  0.144 | -0.03  0.110 | -0.07  *0.034 | -0.01  0.679 | -0.05  *0.023 | 0.04  0.056 | 0.00  0.914 | -0.01  0.722 | -0.53  **0.000 |
| Type of school dependency |  |  |  |  |  |  |  |  |  |  |  |
| Public | 1 | 1 | 1 | 1 | 1 | 1 | 1 | 1 | 1 | 1 | 1 |
| *Subsidized* | 0.02  0.902 | -0.32  0.098 | 0.08  0.562 | -0.09  0.424 | -0.26  0.194 | -0.25  0.213 | 0.15  0.260 | -0.20  0.150 | 0.03  0.873 | -0.02  0.922 | 2.18  *0.032 |
| *Private* | -0.12  0.379 | 0.03  0.877 | -0.16  0.199 | -0.46  **0.000 | -0.71  **0.001 | -0.30  0.105 | -0.40  *0.004 | -0.65  **0.000 | 0.09  0.670 | 0.75  **0.000 | 4.07  *0.000 |
| Educational experiences |  |  |  |  |  |  |  |  |  |  |  |
| Last year self-reported Grade Point Average (GPA) |  |  |  |  |  |  |  |  |  |  |  |
| *Poor* | N/A | 1 | N/A | 1 | N/A | 1 | N/A | 1 | N/A | 1 | 1 |
| *Regular* | N/A | -0.22  0.538 | N/A | -0.43  *0.034 | N/A | -0.69  0.063 | N/A | -0.09  0.705 | N/A | 0.37  0.358 | 4.26  *0.024 |
| *Good* | N/A | -0.24  0.492 | N/A | -0.79  **0.000 | N/A | -1.0  *0.007 | N/A | -0.62  *0.013 | N/A | 0.95  *0.017 | 7.00  *0.000 |
| Academic motivation | 0.06  **0.000 | -0.02  **0.001 | 0.08  **0.000 | -0.02  **0.000 | 0.14  **0.000 | -0.02  **0.000 | 0.04  **0.000 | -0.02  **0.000 | -0.11  **0.000 | 0.04  **0.000 | 0.22  **0.000 |
| Academic self-concept | -0.09  **0.000 | -0.06  **0.000 | -0.06  **0.000 | -0.03  **0.000 | -0.13  **0.000 | -0.07  **0.000 | -0.06  **0.000 | -0.04  **0.000 | 0.07  **0.000 | 0.05  **0.000 | 0.40  **0.000 |
| Sense of belonging | -0.03  *0.002 | -0.05  **0.000 | -0.03  **0.001 | -0.02  **0.000 | -0.06  **0.001 | -0.03  **0.000 | -0.06  **0.000 | -0.04  **0.000 | 0.06  **0.001 | 0.07  **0.000 | 0.40  **0.000 |
| Family functioning |  |  |  |  |  |  |  |  |  |  |  |
| FACES-20 scale | -0.02  **0.000 | -0.02  **0.000 | -0.02  **0.000 | -0.01  **0.000 | -0.02  **0.000 | -0.02  **0.000 | -0.02  **0.000 | -0.02  **0.000 | 0.03  **0.000 | 0.04  **0.000 | 0.25  **0.000 |
| Covid-19 related experiences |  |  |  |  |  |  |  |  |  |  |  |
| Fear to contracting Covid-19 |  |  |  |  |  |  |  |  |  |  |  |
| *No fear* | 1 | 1 | 1 | 1 | 1 | 1 | 1 | 1 | 1 | 1 | 1 |
| *Fear* | 0.25  **0.001 | 0.29  *0.043 | 0.06  0.362 | -0.15  0.066 | 0.17  0.152 | -0.07  0.644 | 0.07  0.416 | -0.06  0.542 | 0.00  0.995 | 0.68  **0.000 | 1.27  0.101 |
| Fear that a family member or friend contracts Covid-19 |  |  |  |  |  |  |  |  |  |  |  |
| *No fear* | 1 | 1 | 1 | 1 | 1 | 1 | 1 | 1 | 1 | 1 | 1 |
| *Fear* | 0.18  *0.025 | 0.66  *0.010 | 0.06  0.435 | -0.05  0.724 | 0.16  0.203 | 0.01  0.974 | 0.14  0.109 | 0.07  0.732 | 0.05  0.717 | 0.77  *0.009 | 0.17  0.902 |
| Socializing online |  |  |  |  |  |  |  |  |  |  |  |
| *No* | 1 | 1 | 1 | 1 | 1 | 1 | 1 | 1 | 1 | 1 | 1 |
| *Yes* | -0.10  0.203 | -0.13  0.347 | -0.07  0.372 | -0.12  0.158 | -0.25  0.061 | -0.11  0.438 | -0.23  *0.011 | -0.39  **0.000 | 0.14  0.271 | 0.50  *0.002 | 1.33  0.076 |
| Doing exercise |  |  |  |  |  |  |  |  |  |  |  |
| *No* | 1 | 1 | 1 | 1 | 1 | 1 | 1 | 1 | 1 | 1 | 1 |
| *Yes* | -0.10  0.165 | -0.24  0.089 | -0.16  *0.025 | -0.08  0.308 | -0.23  0.055 | -0.19  0.185 | -0.23  *0.006 | -0.29  *0.005 | 0.25  *0.036 | 0.34  *0.033 | 1.95  *0.009 |
| Involved in leisure activities |  |  |  |  |  |  |  |  |  |  |  |
| *No* | 1 | 1 | 1 | 1 | 1 | 1 | 1 | 1 | 1 | 1 | 1 |
| *Yes* | -0.14  0.057 | -0.09  0.523 | -0.18  *0.010 | -0.05  0.522 | -0.26  *0.026 | -0.11  0.442 | -0.17  *0.029 | -0.07  0.465 | 0.47  **0.000 | 0.39  *0.016 | 1.94  *0.010 |
| Meditates and prays |  |  |  |  |  |  |  |  |  |  |  |
| *No* | 1 | 1 | 1 | 1 | 1 | 1 | 1 | 1 | 1 | 1 | 1 |
| *Yes* | -0.01  0.853 | -0.59  **0.001 | -0.15  *0.041 | -0.19  0.068 | -0.20  0.105 | -0.45  *0.015 | -0.05  0.517 | -0.18  0.168 | 0.35  *0.004 | 0.84  **0.000 | 2.01  *0.031 |
| Financial problems |  |  |  |  |  |  |  |  |  |  |  |
| *No* | 1 | 1 | 1 | 1 | 1 | 1 | 1 | 1 | 1 | 1 | 1 |
| *Yes* | 0.20  *0.005 | 0.20  0.154 | 0.22  **0.001 | 0.15  0.073 | 0.33  *0.004 | 0.19  0.203 | 0.35  **0.000 | 0.29  *0.005 | 0.38  0.737 | -0.23  0.155 | -2.88  **0.000 |
| Family problems |  |  |  |  |  |  |  |  |  |  |  |
| *No* | 1 | 1 | 1 | 1 | 1 | 1 | 1 | 1 | 1 | 1 | 1 |
| *Yes* | 0.29  **0.000 | 0.65  **0.000 | 0.28  **0.000 | -0.08  0.365 | 0.33  *0.012 | 0.39  *0.012 | 0.38  **0.000 | 0.05  0.666 | -0.28  *0.030 | 0.19  0.256 | -3.38  **0.000 |
| Health problems |  |  |  |  |  |  |  |  |  |  |  |
| *No* | 1 | 1 | 1 | 1 | 1 | 1 | 1 | 1 | 1 | 1 | 1 |
| *Yes* | 0.24  *0.010 | 0.67  **0.000 | 0.16  0.070 | 0.19  0.061 | 0.47  *0.002 | 0.32  0.074 | 0.46  **0.000 | 0.25  *0.044 | 0.02  0.882 | 0.30  0.131 | 33.22  **0.000 |
| Teaching accessibility problems |  |  |  |  |  |  |  |  |  |  |  |
| *No* | 1 | 1 | 1 | 1 | 1 | 1 | 1 | 1 | 1 | 1 | 1 |
| *Yes* | 0.23  *0.003 | 0.23  0.110 | 0.28  **0.000 | 0.13  0.127 | 0.51  **0.000 | 0.30  *0.050 | 0.40  **0.000 | 0.27  *0.013 | -0.14  0.251 | -0.20  0.229 | -3.21  **0.000 |

Note: N/A = not applicable because this variable was not measured in children.

*p≤0.05 and **p≤0.001.
